# Preparation of Hydrochlorothiazide Nanoparticles for Solubility Enhancement †

**DOI:** 10.3390/molecules21081005

**Published:** 2016-08-02

**Authors:** Eliska Vaculikova, Aneta Cernikova, Daniela Placha, Martin Pisarcik, Pavlina Peikertova, Katerina Dedkova, Ferdinand Devinsky, Josef Jampilek

**Affiliations:** 1 Nanotechnology Centre, VSB—Technical University of Ostrava, 17. listopadu 15/2172, 708 33 Ostrava, Czech Republic; eliskavaculikova@seznam.cz (E.V.); pavlina.peikertova@vsb.cz (P.P.); katerina.dedkova@vsb.cz (K.D.); 2 Department of Chemical Drugs, Faculty of Pharmacy, University of Veterinary and Pharmaceutical Sciences, Palackeho 1/3, 612 42 Brno, Czech Republic; cernikova.aneta@gmail.com; 3 IT4 Innovations Centrum Excellence, VSB—Technical University of Ostrava, 17. listopadu 15/2172, 708 33 Ostrava, Czech Republic; 4 Department of Chemical Theory of Drugs, Faculty of Pharmacy, Comenius University, Kalinciakova 8, 832 32 Bratislava, Slovakia; pisarcik@fpharm.uniba.sk (M.P.); devinsky@fpharm.uniba.sk (F.D.); 5 Regional Materials Science and Technology Centre, VSB—Technical University of Ostrava, 17. listopadu 15/2172, 708 33 Ostrava, Czech Republic; 6 Department of Pharmaceutical Chemistry, Faculty of Pharmacy, Comenius University, Odbojarov 10, 832 32 Bratislava, Slovakia

**Keywords:** hydrochlorothiazide, nanoparticles, dynamic light scattering, infrared spectroscopy, scanning electron microscopy, solubility

## Abstract

Nanoparticles can be considered as a useful tool for improving properties of poorly soluble active ingredients. Hydrochlorothiazide (Class IV of the Biopharmaceutical Classification System) was chosen as a model compound. Antisolvent precipitation-solvent evaporation and emulsion solvent evaporation methods were used for preparation of 18 samples containing hydrochlorothiazide nanoparticles. Water solutions of surfactants sodium dodecyl sulfate, Tween 80 and carboxymethyl dextran were used in mass concentrations of 1%, 3% and 5%. Acetone and dichloromethane were used as solvents of the model compound. The particle size of the prepared samples was measured by dynamic light scattering. The selected sample of hydrochlorothiazide nanoparticles stabilized with carboxymethyl dextran sodium salt with particle size 2.6 nm was characterized additionally by Fourier transform mid-infrared spectroscopy and scanning electron microscopy. It was found that the solubility of this sample was 6.5-fold higher than that of bulk hydrochlorothiazide.

## 1. Introduction

**Figure 1:**
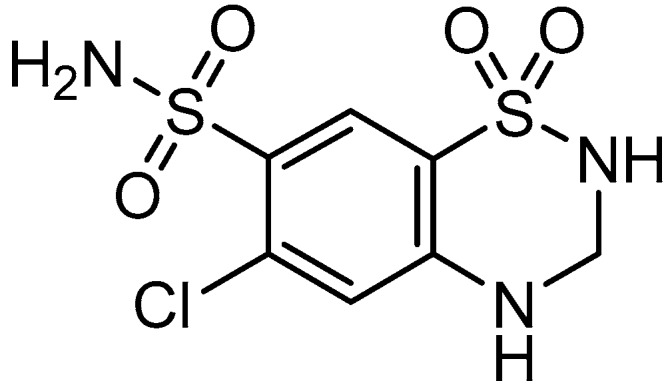
Structure of hydrochlorothiazide.

Hydrochlorothiazide (HCTZ, Figure 1), 6-chloro-3,4-dihydro-2*H*-1,2,4-benzothiadiazine-7-sulfonamide 1,1-dioxide, is a thiazide diuretic that is frequently used in antihypertensive therapy in combination with antihypertensive agents. HCTZ affects the distal renal tubular mechanism of electrolyte reabsorption: it increases excretion of sodium and chloride ions [1]. Due to this increased excretion, it increases diuresis. HCTZ is not a first choice drug for hypertension treatment of patients with diabetes mellitus because of metabolic side effects. HCTZ can cause hyperglycaemia; it can worsen glucose tolerance and cause hypercholesterolemia [2]. It is questionable whether HCTZ nanoparticles (NPs) would have less adverse effects in a range of metabolic side effects. Metabolic side effects of HCTZ are dose-dependent [3,4,5,6,7]. It can be stated that the best balance between effectiveness and side effects is obtained with much smaller doses [8]. This fact was connected with hypokalemia associated with high administrated doses of thiazides (up to 150 mg/day of HCTZ) [8]. Drug NPs have been prepared to provide a possibility of treatment with a lower, but still therapeutic dose [9]. HCTZ belongs to Class IV of the Biopharmaceutical Classification System (BCS) [10]. Drugs of the mentioned class are characterized by poor water solubility and low permeability; this means they are problematic from the point of view of oral bioavailability [11]. Some nanonized drug substances have been used in human treatment. Doses of nanodrugs can be lower than doses of bulk drugs, which results in a lower pill burden [6,12].

The aim of this study was the preparation of stabilized HCTZ NPs via antisolvent precipitation with the following solubility experiment. It was supposed that HCTZ NPs would have improved bioavailability (some HCTZ nanoformulations were published recently [13,14,15,16]). There are two main approaches of NPs preparation: bottom-up and top-down. Top-down techniques are based on milling or high-pressure homogenization. The bottom-up approach is mainly based on precipitation [12,17,18]. This approach was used in this study.

Polar and nonpolar solvents were used in this investigation; therefore, the exact principle of the applied solvent evaporation method is dependent on the water-based system, including or not an aqueous miscible organic solvent. Polar acetone and nonpolar dichloromethane were chosen as the most suitable solvents for easy dissolution of HCTZ; so two different possible mechanisms were used for the NP synthesis. When HCTZ is dissolved in acetone and then mixed with water containing a stabilizer, NPs are formed spontaneously and immediately upon mixing. This method can be called antisolvent precipitation-solvent evaporation, and the procedure is in principle similar to the evaporative precipitation into aqueous solution [19,20] and the liquid antisolvent precipitation [21]. When HCTZ is dissolved in dichloromethane and then mixed with water containing stabilizers, an emulsion (o/w type) is formed; HCTZ is clustered by the excipient, which results in the encapsulation of HCTZ into nano-vesicula. This combination of emulsification and solvent evaporation in NP synthesis is called emulsion solvent evaporation [22,23].

## 2. Results and Discussion

Based on the pilot screening [24,25], excipients such as sodium dodecyl sulfate (Series 1, 2), Tween 80 (Series 3, 4) and carboxymethyl dextran sodium salt (Series 5, 6) were chosen from the group of surfactants. Three water solutions of 1%, 3% and 5% mass concentration were prepared. HCTZ was solved in dichloromethane (Series 1, 3, 5) as a non-polar solvent, and acetone (Series 2, 4, 6) as a polar solvent. Drug solution was added to the solution of excipient under continuous stirring. Organic solvent was evaporated in an ultrasonic bath that was used as a source of complementary energy for NPs preparation. The combination of all excipients with HCTZ provided 18 samples, see Table 1. All prepared samples were measured by dynamic light scattering [26], i.e., the particle size and values of polydispersity index were determined (see Table 1). The suitability of the used surfactant and the organic solvent was analyzed.

The investigated particles showed good particle size stability throughout the light scattering measurements, except for sample **3c** that could not be measured due to crystallization of HCTZ. In the course of the measurements, no significant deviations from the mean values of particle size, which could be a result of possible sample ageing, were observed. In addition, a regular visual check of the samples showed no changes in the sample structure, which was confirmed by the reproducible data obtained by the light scattering method. NPs of size >100 nm were prepared in the case of sodium dodecyl sulfate and carboxymethyl dextran sodium salt in combination with dichloromethane (samples **1b**, **5a**, **5b**) and carboxymethyl dextran sodium salt in combination with acetone (samples **6a**, **6b**). On the other hand, carboxymethyl dextran sodium salt in 5% concentration in combination with dichloromethane or acetone formed samples with the smallest particle sizes (2.6 nm of sample **5c** and 2.8 nm of sample **6c**). Sodium dodecyl sulfate in combination with dichloromethane provided particle size near 20 nm (samples **1a** and **1c**). Other prepared samples contained NPs in the size range from 4.2 to 13.6 nm, mostly near 10 nm.

The dispersity is a measure/degree of the homogeneity/heterogeneity of sizes of particles in a mixture/system. The uniformity of dispersed systems is expressed as polydispersity index (PDI), see Table 1. Low PDI values demonstrate narrow size distribution and the uniformity of samples contrary to PDI ≈ 1, which indicates that samples that have a very broad size distribution, may contain large particles or aggregates and are not suitable for measurements [27,28]. In the prepared NPs of HCTZ, PDI values ranged from 0.128 to 0.396, when the samples **1a** and **5b** (with PDI values 0.654 and 0.470) were eliminated.

**Figure 2:**
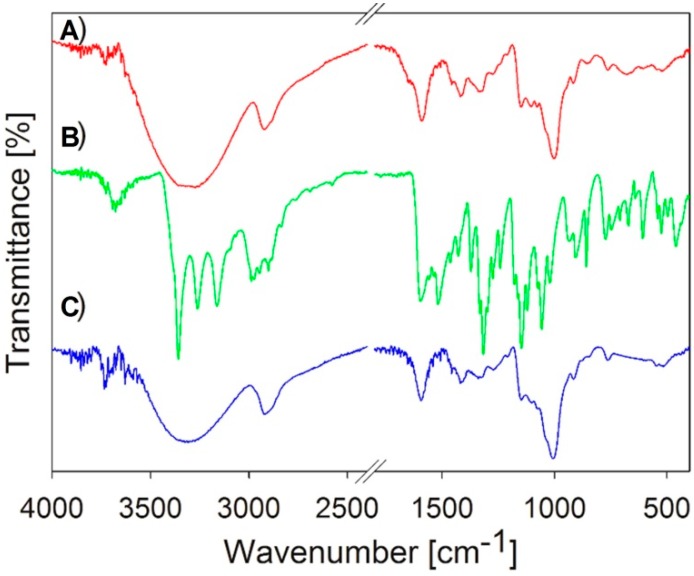
FT-MIR spectra of hydrochlorothiazide stabilized with 5% carboxymethyl dextran sodium salt (sample **5c**, **A**); bulk hydrochlorothiazide (**B**) and carboxymethyl dextran sodium salt (**C**).

**Figure 3:**
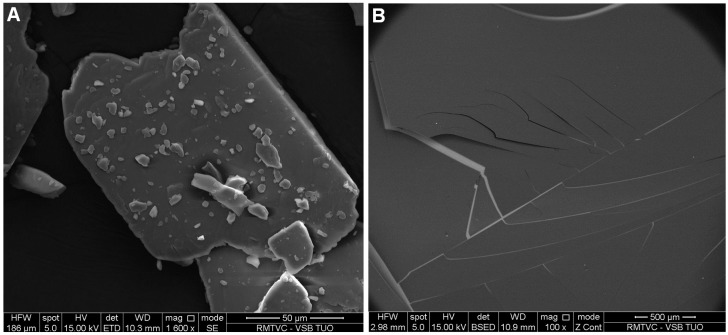
SEM images of bulk hydrochlorothiazide at magnification 1600× (**A**) and hydrochlorothiazide stabilized with 5% carboxymethyl dextran sodium salt (sample **5c**) at magnification 100× (**B**).

Sample **5c** with the smallest particle size and low PDI, where HCZT was stabilized with 5% carboxymethyl dextran sodium salt, was chosen for additional characterization and solubility test. Figure 2 illustrates the Fourier transform mid infrared (FT-MIR) spectra of the starting bulk HCTZ, carboxymethyl dextran sodium salt and HCTZ NPs stabilized with the excipient and thus verifies the composition of sample **5c**. Scanning electron microscopy (SEM) shows bulk HCZT, see Figure 3A, while Figure 3B represents an SEM image showing the surface structure and the morphology of sample **5c**. The surface and structure of nanoparticles were uniform with no roughness.

The solubility of bulk HCTZ (as a control sample) and HCTZ NPs (sample **5c**) was tested; the results of this investigation are presented in Table 2, where the concentrations of HCTZ NPs dissolved in water with pH 6 were determined by HPLC in comparison with bulk HCTZ. The dissolved amount of bulk HCTZ was 16.6 µg/mL, while that of HCTZ NPs was 107.9 µg/mL, i.e., the application of NPs led to a significant, 6.5-fold increase of the solubility of HCTZ.

Water soluble, biodegradable, biocompatible and nontoxic carboxymethyl dextran has been widely used as a stabilizer and a nanocarrier for drug delivery [29,30,31,32,33,34,35,36]. Based on the experiments, it can be stated that carboxymethyl dextran sodium salt can be considered as a versatile stabilizer for a variety of drugs from classes II–IV of the Biopharmaceutical Classification System, for example, lipophilic steroids [24], highly hydrophilic risedronate sodium [18], basic cimetidine [37], acid candesartan cilexetil and calcium salt of atorvastatin [25]. In general, it provided stable NP samples with narrow particle size distribution in concentrations of 3% and 5% in dichloromethane rather than in acetone [17,24,25,37]. This may be connected with properties of non-polar dichloromethane (with dipole moment 1.60 D in contrast to polar aprotic acetone with dipole moment 2.88 D [38]) and hydrophilic charged polymeric carboxymethyl dextran sodium salt and their (non)bonding interactions with used molecules of drugs [30,31,32,33,34]. Despite its toxicity, dichloromethane has been one of the most widely used solvents of drugs of intrinsic emulsified phase [29,39] dispersed in aqueous solution of carboxymethyl dextran sodium salt. The different mechanisms of generation of NPs in comparison with the antisolvent precipitation process in the presence of polar acetone seems to be another important parameter affecting the particle size and the homogeneity of the samples. Nevertheless, it can be stated that the “net” formed by 5% concentration of carboxymethyl dextran sodium salt prevents aggregation/agglomeration of drug substance particles.

## 3. Experimental Section

### 3.1. General Procedure for Preparation of Nanoparticles

The model compound hydrochlorothiazide (HCTZ) and the excipients sodium dodecyl sulfate, Tween 80 and carboxymethyl dextran sodium salt were purchased from Sigma-Aldrich (St. Louis, MO, USA). All compounds were of analytical grade. H_2_O-HPLC-Mili-Q Grade was used as a solvent of the excipients. Each excipient (0.1, 0.3 or 0.5 g) was dissolved in water (10 mL), and three solutions with mass concentrations 1%, 3% and 5% were prepared. HCTZ (0.1 g) was dissolved in dichloromethane or acetone (10 mL), i.e., 1% solutions were prepared. The solution of HCTZ in dichloromethane/acetone was slowly dropped (2 mL/min) to the aqueous solutions of excipients, and the solutions were stirred at 600 rpm for 15 min at 25 °C, after which the mixtures were transferred to the ultrasonic bath in the fume chamber, where they were mixed again for 20 min for homogenization of the samples. Finally, the solvent was evaporated.

### 3.2. Particle Size Measurement

The particle size was determined using a Brookhaven dynamic light scattering system BI 9000 (Brookhaven Instruments Corporation, Holtsville, NY, USA) with a goniometer SM-200 and an argon gas laser (Lexel 95, wavelength 514.5 nm). Scattered intensity was registered at scattering angle 90° and temperature 25 °C. All the samples were dispersed by sonication and additionally filtered directly before the measurement through syringe filters with 0.45 μm pore size to remove mechanical impurities. Five independent recordings of the autocorrelation function were done for each investigated excipient concentration. The particle size was calculated from the translational diffusion coefficient using the Stokes-Einstein formula. The translational diffusion coefficient was obtained based on the cumulant expansion of the autocorrelation function up to the second cumulant. The presented particle sizes are reported as the mean values taken of the set of five independent measurements. The results are summarized in Table 1.

### 3.3. FT-IR Analysis

Fourier transform mid infrared (FT-MIR) spectra (Figure 2) were measured for confirmation of the sample composition. The attenuated total reflectance (ATR) technique was used with a diamond crystal. FT-MIR spectra were recorded by a Nicolet 7000 FT-IR spectrometer (Thermo Scientific, West Palm Beach, FL, USA) in the range 4000–400 cm^−1^ with the resolution of 4 cm^−1^ and 32 scans.

### 3.4. Scanning Electron Microscopy

The morphology of the sample was studied using scanning electron microscopy (SEM). The sample was attached to a microscopic glass, dried at 25 °C and then sputter-coated with thin film of gold. An electron microscope Quanta 450FEG (FEI, Hillsboro, OR, USA) was used for imaging of the sample at the following working conditions: accelerating voltage 10.0 kV, working distance 8.0 mm and probe current 100 mA. Structures of the samples are illustrated in Figure 3.

### 3.5. Solubility Test

Two beakers filled with 10 mL of phosphate buffer (pH 6) were placed on magnetic stirrers and tempered to 36 °C [40,41,42]. Dry powders of bulk HCTZ or sample **5c** were separately added to beakers with buffer under continuous stirring (250 rpm). The beakers were covered by Parafilm^®^. When the turbidity occurred, the content of the beaker was stirred for additional 24 h. If the turbidity did not disappear, the samples were centrifuged and analyzed. Analysis of samples was performed using a high performance liquid chromatograph Agilent 1200 Series (Agilent Technologies, Santa Clara, CA, USA) equipped with a DAD, a quarter pump, and an automatic injector. The HPLC separation process was monitored and evaluated by the ChemStation software. A chromatographic column Gemini 5 µm C6-Phenyl 110A, 4.6 mm × 250 mm (Phenomenex^®^, Torrance, CA, USA) was used. Isocratic elution by a mixture of MeOH p.a. (35%) and formic buffer (pH 4.0; 65%) as a mobile phase was used. The total flow of the column was 1.0 mL/min, injection 10 μL, column temperature 40 °C, and sample temperature 22 °C. The detection wavelength of 273 nm was chosen. The retention time of HCTZ was 4.19 ± 0.05 min, the limit of detection (LOD) was 0.01 µg/mL, and the limit of quantification (LOQ) of HCTZ was 0.04 µg/mL. The results are presented in Table 2.

## 4. Conclusions

Eighteen samples of HCTZ NPs were prepared by the antisolvent precipitation-solvent evaporation and the emulsion solvent evaporation techniques. Three different excipients were used as stabilizers in 1%, 3%, and 5% aqueous mass concentration. Dynamic light scattering was used for particle size analysis. The polydispersity index (PDI) was determined. Low PDI values of the samples confirmed narrow size distribution and the uniformity of the prepared systems. Sample **5c** of HCTZ NPs stabilized with 5% concentration of carboxymethyl dextran sodium salt (particle size 2.3 nm, PDI 0.212) was chosen as the sample with the smallest particle size for following characterization and analysis. SEM was used for surface identification, and FT-MIR spectroscopy was used for verification of the sample composition. Finally, the solubility test confirmed the enhanced solubility of HCTZ NPs in comparison with the solubility of bulk HCTZ (6.5-fold higher).

## Figures and Tables

**Table 1:** Composition of samples (dichloromethane Series 1, 3, 5; acetone Series 2, 4, 6), concentration (%) of individual excipients in water samples relative to hydrochlorothiazide, particle size (nm) and polydispersity index (PDI) of hydrochlorothiazide samples expressed as mean ± SD (*n* = 5 independent measurements). (SDS = sodium dodecyl sulfate, TW = Tween 80, DCM = dichloromethane, CMD = carboxymethyl dextran sodium salt, AC = acetone, n.d. = immeasurable due to crystallization)

Sample	Excipient/Concentration (%)	Particle Size (nm)	PDI
**1a**	SDS/DCM/1	24.5 ± 3.6	0.654 ± 0.073
**1b**	SDS/DCM/3	102.2 ± 17.6	0.384 ± 0.021
**1c**	SDS/DCM/5	21.0 ± 1.2	0.356 ± 0.011
**2a**	SDS/AC/1	9.6 ± 0.4	0.302 ± 0.014
**2b**	SDS/AC/3	4.2 ± 0.6	0.300 ± 0.027
**2c**	SDS/AC/5	7.6 ± 1.2	0.271 ± 0.012
**3a**	TW/DCM/1	10.5 ± 0.5	0.177 ± 0.020
**3b**	TW/DCM/3	13.6 ± 0.7	0.189 ± 0.008
**3c**	TW/DCM/5	n.d.	n.d.
**4a**	TW/AC/1	10.2 ± 0.1	0.154 ± 0.007
**4b**	TW/AC/3	9.3 ± 0.3	0.128 ± 0.057
**4c**	TW/AC/5	9.9 ± 0.1	0.157 ± 0.010
**5a**	CMD/DCM/1	107.0 ± 51.4	0.396 ± 0.025
**5b**	CMD/DCM/3	221.5 ± 28.5	0.470 ± 0.034
**5c**	CMD/DCM/5	2.6 ± 0.2	0.212 ± 0.140
**6a**	CMD/AC/1	493.2 ± 155.9	0.368 ± 0.075
**6b**	CMD/AC/3	510.7 ± 33.7	0.396 ± 0.055
**6c**	CMD/AC/5	2.8 ± 1.2	0.312 ± 0.040

**Table 2:** Results of dissolution test.

Sample	HPLC Determined Conc. (µg/mL)	R
**bulk HCTZ (control sample)**	16.6	–
**HCTZ NPs (sample 5c)**	107.9	6.5
